# Association between night sleep latency and hypertension: A cross-sectional study

**DOI:** 10.1097/MD.0000000000031250

**Published:** 2022-10-21

**Authors:** Xia Zhong, Fuyue Gou, Huachen Jiao, Dongsheng Zhao, Jing Teng

**Affiliations:** a Department of First Clinical Medical College, Shandong University of Traditional Chinese Medicine, Jinan, PR China; b Department of Cardiology, Affiliated Hospital of Shandong University of Traditional Chinese Medicine, Jinan, PR China.

**Keywords:** gender, hypertension, PSQI, risk, sleep latency

## Abstract

Sleep disorders have been shown to increase the risk of hypertension, while the relationship between night sleep latency and hypertension is less well-known. We aimed to investigate the association between night sleep latency and hypertension, as well as related sleep factors by gender in the Chinese population. We conducted a cross-sectional study of the relationship between night sleep latency and hypertension. The sample size included 619 consecutive hospitalized patients (M/F: 302/317, 64.01 ± 12.27 years). *T* test, Chi-square test, and ANOVA were performed to analyze baseline data and intergroup comparisons. Spearman correlation analysis was performed to find the interrelationships. Multivariate logistic regression analysis was performed to adjust for covariables. The findings showed hypertension patients had longer night sleep latency (*P* < .001). After adjusting for confounding factors, night sleep latency was positively correlated with hypertension in both men and women (odds ratio: 1.065, 95% confidence interval: 1.044–1.087). Spearman correlation analysis suggested that night sleep latency was positively correlated with systolic blood pressure (*r* = 0.186, *P* < .001), diastolic blood pressure (*r* = 0.136, *P* < .001), sleep initiation time (*r* = 0.091, *P* = .023), and global Pittsburg Sleep Quality Index score (*r* = 0.371, *P* < .001), was negatively correlated with sleep duration (*r* = −0.186, *P* < .001), sleep time on weekdays (*r* = −0.183, *P* < .001), and sleep time on weekends (*r* = −0.179, *P* < .001). Longer night sleep latency was associated with an increased risk of hypertension in men and women, which might involve the pathological progression of hypertension along with other sleep factors.

## 1. Introduction

Hypertension is the increasing prevalence of the disease worldwide, affecting about 35% of adults and 90% of the elderly population, and contributes to increased cardiovascular morbidity and mortality 2- to 4-fold.^[1–4]^ It is statistical that the lifetime risk of hypertension in the United States is about 90%.^[5,6]^ Hypertension represents a heavy financial burden on the healthcare system^[3,7]^ and its prevalence is expected to increase by about 60% in 2025.^[1]^ To date, although the awareness and treatment for hypertension have been improved, the control rate of the hypertension population remains low, lower than 20% to 30% in some Western countries.^[8,9]^ There is growing evidence that managing the overall risk of patients with hypertension is more valuable than just focusing on blood pressure measurements.^[10]^ Therefore, it is essential to explore the potential risk factors of hypertension and establish effective prevention and risk management strategies.

Sleep is an essential unit in the human lifestyle. In recent years, a growing number of studies have focused on the relationship between sleep and hypertension. The available evidence suggests that poor sleep conditions may be an important risk factor involved the pathophysiological abnormalities of hypertension and related comorbid states.^[11–13]^ As we all know, hypertension is closely related to the imbalance of the autonomic nervous system. Sleep plays an important role in maintaining homeostasis to regulate the stress system.^[14,15]^ A putative link between sleep disorders causing sympathetic and vagal imbalances and ultimately cardiac autonomic dysfunction has been reported.^[16,17]^ Thus, some scholars have proposed that poor sleep quality may be involved in the development of hypertension through the activation of the sympathetic nervous system and pro-inflammatory pathways.^[18–20]^

Although current evidence suggests that there appears to be a strong link between sleep duration and hypertension, the relationship between sleep quality and hypertension needs to be further explored.^[21–24]^ The differences between men and women in sleep have been found in past reports. Unfortunately, due to differences in geography, population, confounding factors, or methods, the regularity and underlying mechanism of sex differences between sleep quality and hypertension remain unclear. The current evidence suggests that the association between poor sleep quality and hypertension may be stronger in women than in men.^[25,26]^ However, several studies have reported different results. A cross-sectional study suggested that poor sleep quality was associated with an increased prevalence of hypertension only in men.^[27]^ Another study also reported that there was no significant relationship between sleep quality and arterial blood pressure.^[28]^

The Pittsburg Sleep Quality Index (PSQI) questionnaire is a popular and validated tool to evaluate sleep quality, which involves subjective sleep quality, sleep duration, sleep latency, sleep disturbances, habitual sleep efficiency, daytime dysfunction, and use of sleeping pills.^[29]^ From a clinical point of view, the difficulty of falling asleep is the most common sleep complaint. Sleep latency is the specific characteristic of sleep patterns that might be independently related to hypertension. Briefly, sleep latency is the amount of time it takes a person to fall asleep in bed, which is an important marker for assessing sleep quality.

To our knowledge, there have been few previous studies exploring the influence of night sleep latency on hypertension by gender in the Chinese population. The objective of this study was to investigate the association between sleep latency and the risk of hypertension, as well as hypertension-related sleep factors by gender in the Chinese population. We hypothesized that long sleep latency would be related to the higher risk of hypertension, and involved in the pathological development of hypertension along with several related sleep factors.

## 2. Methods

### 2.1. Study design and participants

We conducted a cross-sectional study based on the Chinese population. All participants were recruited from April 2020 to December 2020 in 10 different regions of Shandong province, including Jinan, Qingdao, Zibo, Yantai, Dongying, Liaocheng, Qixia, Penglai, Laiyang, and Haiyang. We screened hospitalized patients aged 27 to 92 years with complete clinical data by random lottery method. Participants with secondary hypertension, heart failure, coronary heart disease, arrhythmias, valvular abnormalities, structural heart disease, cardiac surgery, malignant tumors, severe liver or kidney dysfunction, hyperthyroidism, cognitive or memory impairment, pregnant women, night shift status, and individuals who are taking beta blockers in the last 2 weeks were excluded. Before the study, 10 qualified investigators were given standardized training. The samples collected consisted mainly of physical examination and questionnaire interview, including age, sex, body mass index (BMI), smoking, alcohol consumption, educational level, systolic blood pressure (SBP), diastolic blood pressure (DBP), sitting time, and sleep parameters. Participants answered survey questions face-to-face with the assistance of investigators and family members at the hospital. At baseline, 619 participants were examined after 34 samples with missing or invalid data were excluded. This study followed the principles of the Helsinki Declaration, was approved by the Medical Research Ethics Committee of The Affiliated Hospital of Shandong University of Traditional Chinese Medicine, and obtained the informed consent of all participants at a visit.

### 2.2. Assessment of blood pressure

All participants’ blood pressure measurements were taken by doctors in the ward when the patients woke up in the morning. Participants avoided drinking tea, coffee, or alcohol, smoking, and exercising for 30 minutes before taking their blood pressure. All blood pressure measurements were taken during one visit. SBP and DBP were measured while participants were supine or sitting. SBP and DBP measurements were taken three times at 2-minute intervals at each visit, the average of the three measurements was calculated and obtained.^[30,31]^ Hypertension was defined as SBP ≥ 140 mm Hg and/or DBP ≥ 90 mm Hg, or the use of antihypertensive drugs.^[32,33]^

### 2.3. Sleep parameters

The sleep parameters of all participants were evaluated by PSQI developed by Buysse et al^[29]^ PSQI is a self-assessment questionnaire consisting of 19 items and 7 component scores. PSQI score ≤5 is classified as good sleep quality, ≥6 is classified as poor sleep quality.^[34]^ According to the questionnaire, we obtained relevant sleep parameters of all participants, including sleep duration, sleep initiation time, sleep latency, sleep duration on weekdays and weekends, etc. The night sleep latency was divided into four categories: ≤10 minutes, 10 to ≤20 minutes, 20 to ≤30 minutes, and >30 minutes. The questions set in the questionnaire are as follows: How many minutes did it take you to sleep at night in the past 30 days?“ (Night sleep latency) “How many hours did you sleep at night in the past 30 days?” (Night sleep duration) “What time do you usually start to sleep at night in the past 30 days?” (Sleep initiation time) Like this, we obtained relevant sleep parameters for all participants in this question-and-answer format.

### 2.4. Statistical analysis

In this study, we performed all data analysis and graphing using IBM SPSS version 26.0 (SPSS Inc., Chicago, IL) and GraphPad Prism version 9.0.0 (Windows). Continuous data were expressed as mean ± standard deviation (SD) and compared with Student’s *T* test and ANOVA. Categorical data were expressed as n (%) and compared with chi-square. Spearman correlation analysis was used as a scatter plot to evaluate the interrelationships of variables. Multivariate regression analysis was performed using odds ratios (ORs) and 95% confidence intervals (95% CI) to adjust for covariates. *P* < .05 was considered significant, double-tailed test.

## 3. Results

### 3.1. Demographic characteristics of participants

**Figure 1.**
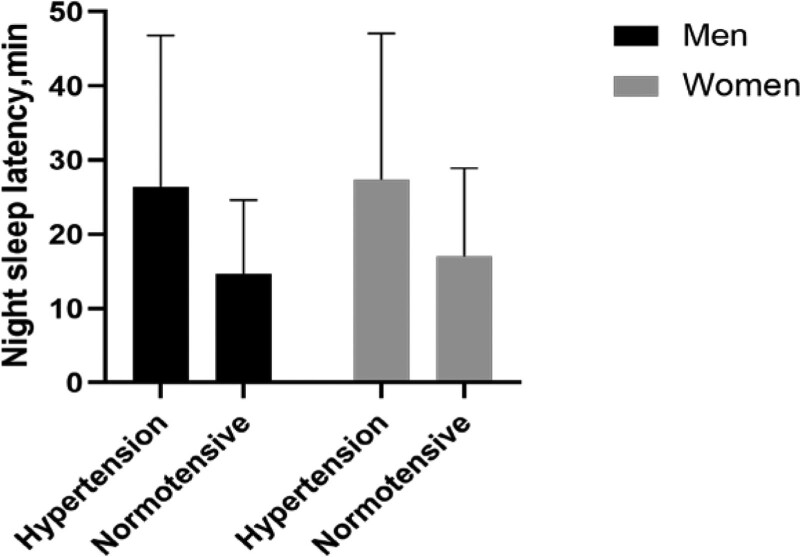
Night sleep latency in the hypertension group and normotensive group by gender. Compared with controls, night sleep latency of the hypertension group was significantly higher in the men (26.32 ± 20.48 min vs 14.65 ± 9.94 min, *P* < .001) and women (27.41 ± 19.65 min vs 17.02 ± 11.88 min, *P* < .001).

As shown in Table 1, among the 619 participants, there were 447 patients with hypertension (72.21%), including 224 males and 223 females, with an average age of 63.47 ± 11.94 years. Compared with controls, patients with hypertension were more likely to be higher SBP, DBP, global PSQI score, and the PSQI sub-component scores, shorter night sleep duration, and longer night sleep latency (*P* < .05). As shown in Figure 1, compared with controls, night sleep latency of the hypertension group was significantly higher in the men (26.32 ± 20.48 minutes vs 14.65 ± 9.94 minutes, *P* < .001) and women (27.41 ± 19.65 minutes vs 17.02 ± 11.88 minutes, *P* < .001).

**Table 1:** Demographic characteristics of participants.

Variables	Normotensive (n = 172)	Hypertension (n = 447)	*P* value
Age, yr	65.39 ± 13.02	63.47 ± 11.94	.081
Gender			.288
Men	78 (45.3)	224 (50.1)	
Women	94 (54.7)	223 (49.9)	
BMI, kg/m^2^	25.69 ± 4.24	25.55 ± 3.76	.689
Smoking			.626
Yes	47 (27.3)	131 (70.7)	
No	125 (72.7)	316 (29.3)	
Drinking			.299
Yes	48 (27.9)	144 (32.2)	
No	124 (72.1)	303 (67.8)	
Educational levels			.219
Primary school or below	42 (24.4)	139 (31.1)	
Junior or Senior high school	109 (63.4)	251 (56.2)	
University or above	21 (12.2)	57 (12.8)	
SBP, mm Hg	126.64 ± 8.97	156.19 ± 17.86	<.001*
DBP, mm Hg	76.58 ± 7.18	90.07 ± 10.45	<.001*
Sitting time, min			
On weekdays	230.38 ± 129.98	233.56 ± 147.87	.793
On weekends	176.48 ± 146.52	180.44 ± 154.50	.772
Sleep time, min			
On weekdays	405.35 ± 99.76	417.13 ± 12.09	.124
On weekends	413.55 ± 103.34	420.63 ± 132.48	.482
Night sleep duration, h	6.92 ± 1.09	6.66 ± 1.52	.018*
Night sleep initiation time, h	21.54 ± 0.80	21.61 ± 1.07	.378
Night sleep latency, min	15.94 ± 11.08	26.86 ± 20.05	<.001*
Global PSQI score, points	7.66 ± 3.89	9.92 ± 3.36	<.001*
Subjective sleep quality score, points	1.04 ± 0.78	1.21 ± 0.67	.010*
Sleep latency score, points	1.31 ± 0.97	1.54 ± 0.81	.006*
Sleep duration score, points	1.02 ± 0.79	1.19 ± 0.64	.012*
Habitual sleep effificiency score, points	0.75 ± 1.00	1.04 ± 0.96	.001*
Sleep disturbance score, points	1.21 ± 0.52	1.34 ± 0.51	.005*
Use of sleep medication score, points	0.76 ± 1.04	1.81 ± 0.92	<.001*
Daytime dysfunction score, points	1.53 ± 1.05	1.77 ± 0.95	.006*

Data were presented as mean ± SD or n (%).

BMI = body mass index, DBP = diastolic blood pressure, PSQI = Pittsburgh Sleep Quality Index, SBP = systolic blood pressure.

* Statistically significant value (*P* < .05).

### 3.2. Correlation between night sleep latency and blood pressure in patients with hypertension

**Figure 2.**
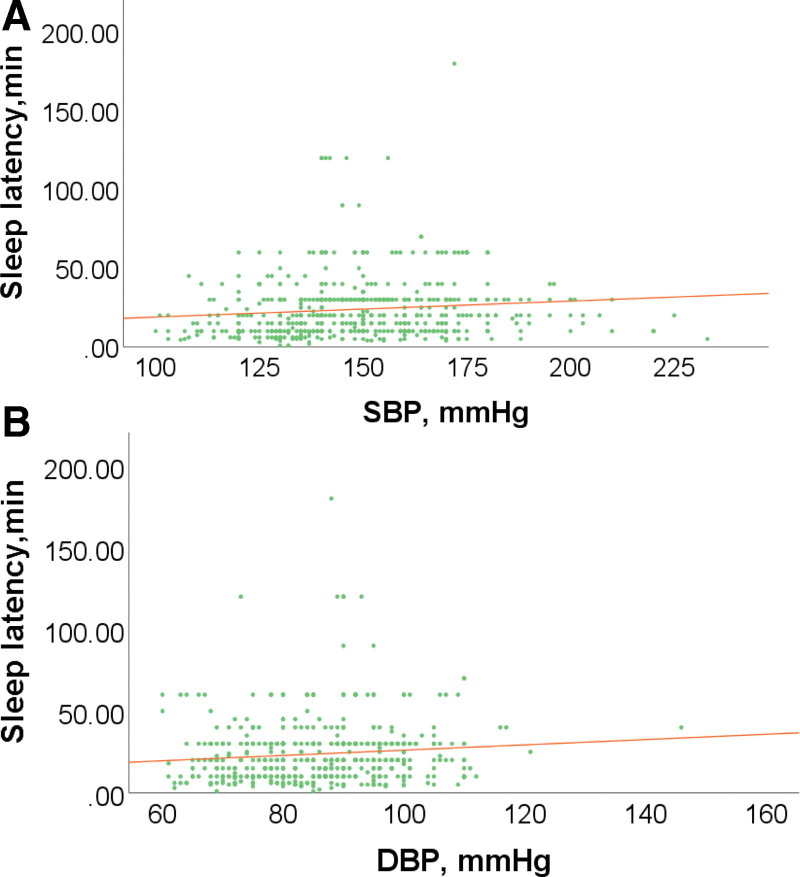
The scatter plots showed the correlation between night sleep latency and blood pressure in patients with hypertension. (A) Correlation between night sleep latency and SBP in patients with hypertension (*r* = 0.186, *P* < .001). (B) Correlation between night sleep latency and DBP in patients with hypertension (*r* = 0.136, *P* < .001). DBP = diastolic blood pressure, SBP = systolic blood pressure.

As shown in Figure 2, the current results showed that night sleep latency was positively correlated with SBP (*r* = 0.186, *P* < .001, Fig. 2A) and DBP (*r* = 0.136, *P* < .001, Fig. 2B).

### 3.3. Correlation between night sleep latency and related sleep factors in patients with hypertension

**Figure 3.**
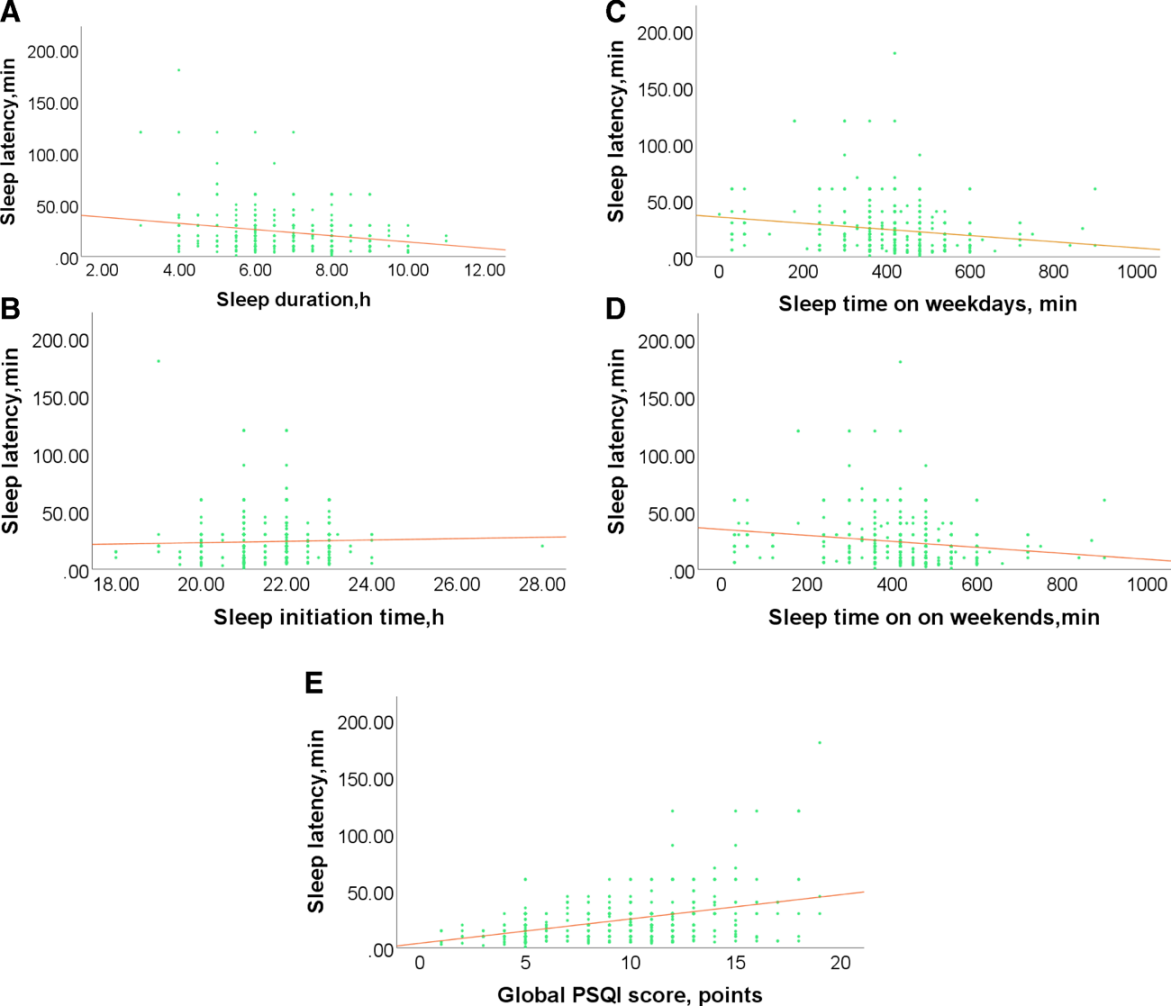
The scatter plots showed the correlation between night sleep latency and related sleep factors in patients with hypertension. (A) Correlation between night sleep latency and sleep duration (*r* = −0.186, *P* < .001). (B) Correlation between night sleep latency and sleep initiation time (*r* = 0.091, *P* = .023). (C) Correlation between night sleep latency and sleep time on weekdays (*r* = −0.183, *P* < .001). (D) Correlation between night sleep latency and sleep time on weekends (*r* = −0.179, *P* < .001). (E) Correlation between night sleep latency and global PSQI score (*r* = 0.371, *P* < .001). PSQI = Pittsburgh Sleep Quality Index.

Figure 3 showed that night sleep latency was negatively correlated with sleep duration (*r* = −0.186, *P* < .001, Fig. 3A), sleep time on weekdays (*r* = −0.183, *P* < .001, Fig. 3C), sleep time on weekends (*r* = −0.179, *P* < .001, Fig. 3D), and was positively correlated with sleep initiation time (*r* = 0.091, *P* = .023, Fig. 3B), global PSQI score (*r* = 0.371, *P* < .001, Fig. 3E).

### 3.4. Associations between night sleep latency and hypertension

As shown in Table 2, after adjusting for age, educational levels, BMI, smoking, drinking, sitting time on weekdays, sitting time on weekends, night sleep latency was related to hypertension (OR = 1.064, 95% CI: 1.045–1.083, *P* < .001). After adjusting for sleep time on weekdays, sleep time on weekends, night sleep duration, sleep initiation time, sleep latency, PSQI score, subjective sleep quality score, sleep latency score, sleep duration score, habitual sleep efficiency score, sleep disturbance score, use of sleep medication score, daytime dysfunction score., night sleep latency was still associated with hypertension (OR = 1.062, 95% CI: 1.041–1.083, *P* < .001). Further after adjusting for all confounding factors, night sleep latency remained as an independent positive correlation with hypertension (OR = 1.065, 95% CI: 1.044–1.087, *P* < .001). Moreover, night sleep latency was positively correlated with hypertension in both men and women (*P* < .001).

**Table 2:** ORs (95% CIs) of night sleep latency for hypertension.

	Total		Men		Women	
	OR (95% CI)	*P* value	OR (95% CI)	*P* value	OR (95% CI)	*P* value
Model 1	1.061 (1.043–1.079)	<.001*	1.074 (1.044–1.105)	<.001*	1.053 (1.030–1.076)	<.001*
Model 2	1.064 (1.045–1.083)	<.001*	1.080 (1.048–1.112)	<.001*	1.054 (1.031–1.078)	<.001*
Model 3	1.062 (1.041–1.083)	<.001*	1.066 (1.030–1.103)	<.001*	1.060 (1.033–1.087)	<.001*
Model 4	1.065 (1.044–1.087)	<.001*	1.076 (1.037–1.116)	<.001*	1.063 (1.036–1.092)	<.001*

Model 1: crude, no adjustment.

Model 2: adjusting for age, educational levels, BMI, smoking, drinking, sitting time on weekdays, sitting time on weekends.

Model 3: adjusting for sleep time on weekdays, sleep time on weekends, night sleep duration, sleep initiation time, sleep latency, PSQI score, subjective sleep quality score, sleep latency score, sleep duration score, habitual sleep efficiency score, sleep disturbance score, use of sleep medication score, daytime dysfunction score.

Model 4: adjusting for all these factors.

BMI = body mass index, OR = odd ratio, PSQI = Pittsburgh Sleep Quality Index.

* Statistically significant value (*P* < .05).

### 3.5. Prevalence and ORs (95% CI) for stratified analysis of night sleep latency for hypertension

As shown in Table 3, night sleep latency was divided into four categories: ≤10 minutes, 10 to ≤20 minutes, 20 to ≤30 minutes, and >30 minutes. Compared with the reference group (night sleep latency ≤10 minutes), the risk of hypertension was significantly higher among those with night sleep latency of >10 minutes after adjusting for confounding factors (10 to ≤20 minutes: OR = 2.59, 95% CI = 1.58–4.23; 20 to ≤30 minutes: OR = 6.67, 95% CI = 3.67–12.11; >30 minutes: OR = 2.99, 95% CI = 1.50–5.97), and the prevalence was significantly higher (10 to ≤20 minutes: 72.39%; 20 to ≤30 minutes: 88.82%; >30 minutes: 82.42%).

**Table 3:** Prevalence and ORs (95% CI) for stratified analysis of night sleep latency for the risk of hypertension.

Variables	Night sleep latency (min)							
	≤10 (n = 195)		10 to ≤20 (n = 163)		20 to ≤30 (n = 170)		>30 (n = 91)	
Total	95% CI	*P* value	95% CI	*P* value	95% CI	*P* value	95% CI	*P* value
Prevalence (%)	52.82		72.39		88.82		82.42	
Model 1	Reference	–	2.34 (1.50–3.65)	<.001*	7.10 (4.08–12.35)	<.001*	4.19 (2.28–7.70)	<.001*
Model 2	Reference	–	2.59 (1.58–4.23)	<.001*	6.67 (3.67–12.11)	<.001*	2.99 (1.50–5.97)	.002*
Men	102	–	81		79		40	
Prevalence (%)	28.21		37.42		42.35		39.56	
Model 1	Reference		2.61 (1.38–4.93)	.003*	8.79 (3.69–20.94)	<.001*	7.69 (2.55–23.20)	<.001*
Model 2	Reference	–	2.18 (1.04–4.57)	.040*	9.08 (3.29–25.01)	<.001*	3.95 (1.08–14.42)	.038*
Women		–	82		91	51		
Prevalence (%)	24.62		34.97		46.47		42.86	
Model 1	Reference		2.14 (1.15–3.98)	.017*	6.17 (2.97–12.82)	<.001*	3.05 (1.42–6.54)	.004*
Model 2	Reference	–	2.77 (1.36–5.64)	.005*	5.69 (2.60–12.44)	<.001*	2.44 (1.03–5.76)	.042*

Model 1: crude, no adjustment.

Model 2: adjusting for age, educational levels, marital status, smoking, drinking, sitting time on weekdays, sitting time on weekends, sleep time on weekdays, sleep time on weekends, BMI, global PSQI score, sleep initiation time, and sleep duration.

BMI = body mass index, OR = odd ratio, PSQI = Pittsburgh Sleep Quality Index.

* Statistically significant value (*P* < .05).

### 3.6. Association of night sleep latency categories and related sleep factors in men and women hypertension patients

As shown in Table 4, longer night sleep latency had a higher global PSQI score, subjective sleep quality score, sleep latency score, daytime dysfunction score in men and women hypertension patients (*P* < .001). In addition, longer night sleep latency had shorter sleep time on weekdays and sleep time on weekends in men hypertension patients (*P* < .05), longer night sleep latency had higher sleep duration score in women hypertension patients.

**Table 4:** Association of night sleep latency categories and related sleep factors in men and women hypertension patients.

Variables	Men (n = 224)				Women (n = 223)			
	≤15 min	15–30 min	≥30 min	*P* value	≤15 min	15–30 min	≥30 min	*P* value
Number, n	75	47	102		70	40	113	
Sleep duration, h	6.87 ± 1.59	6.82 ± 1.54	6.44 ± 1.34	.076	6.66 ± 1.51	6.80 ± 1.55	6.50 ± 1.54	.531
Sleep initiation time, h	21.55 ± 1.09	21.60 ± 1.53	21.80 ± 0.91	.215	21.39 ± 1.07	21.26 ± 1.12	21.65 ± 0.88	.056
Sleep time on weekdays, min	449.45 ± 114.99	427.66 ± 102.50	392.45 ± 126.02	<.001*	432.14 ± 119.78	398.25 ± 168.75	400.22 ± 135.78	.265
Sleep time on weekends, min	452.97 ± 117.01	430.21 ± 110.37	396.37 ± 131.25	.002*	434.29 ± 126.25	406.50 ± 168.06	402.04 ± 139.87	.310
Global PSQI score, points	8.83 ± 3.34	9.36 ± 2.76	10.54 ± 3.19	<.001*	9.31 ± 3.46	9.03 ± 2.74	11.32 ± 3.37	<.001*
Subjective sleep quality score, points	0.99 ± 0.63	1.00 ± 0.55	1.36 ± 0.67	<.001*	1.10 ± 0.66	1.02 ± 0.53	1.46 ± 0.70	<.001*
Sleep latency score, points	0.97 ± 0.84	1.34 ± 0.52	1.82 ± 0.74	<.001*	1.27 ± 0.85	1.40 ± 0.63	1.96 ± 0.67	<.001*
Sleep duration score, points	1.03 ± 0.52	1.06 ± 0.64	1.24 ± 0.66	.054	1.14 ± 0.55	1.08 ± 0.57	1.37 ± 0.73	.015*
Habitual sleep efficiency score, points	0.93 ± 0.79	1.04 ± 0.91	0.98 ± 0.94	.799	1.11 ± 0.96	0.85 ± 0.86	1.17 ± 1.10	.232
Sleep disturbance score, points	1.17 ± 0.38	1.34 ± 0.56	1.31 ± 0.49	.080	1.31 ± 0.50	1.40 ± 0.55	1.45 ± 0.53	.216
Use of sleep medication score, points	1.71 ± 1.10	1.96 ± 0.78	1.88 ± 0.88	.306	1.67 ± 0.88	1.78 ± 0.86	1.83 ± 0.91	.499
Daytime dysfunction score, points	1.57 ± 0.98	1.57 ± 0.93	1.89 ± 0.88	.038*	1.67 ± 1.06	1.50 ± 0.88	2.03 ± 0.90	.003*

PSQI = Pittsburgh Sleep Quality Index.

* Statistically significant value (*P* < .05).

## 4. Discussion

The main finding of our study was to determine the association between night sleep latency and hypertension in a cross-sectional study based on the Chinese population. The results of our study showed that longer night sleep latency in hypertension patients and an independent positive correlation between night sleep latency and hypertension in men and women. Further results suggested that night sleep latency was positively correlated with SBP, DBP, sleep initiation time, and global PSQI score was negatively correlated with sleep duration, sleep time on weekdays, and sleep time on weekends. Additionally, we also found that longer night sleep latency had higher global PSQI score, subjective sleep quality score, sleep latency score, daytime dysfunction score in men and women hypertension patients, had shorter sleep time on weekdays and sleep time on weekends in men hypertension patients, had higher sleep duration score in women hypertension patients. These significant findings may contribute to the understanding of the relationship between night sleep latency and hypertension, as well as hypertension-related sleep factors.

It has been demonstrated that sleep disorder or poor sleep quality was a significant risk factor for hypertension.^[35,36]^ Recently, although night sleep duration, sleep initiation time, and global PSQI score have been studied extensively,^[22,23,37,38]^ other significant measures of sleep quality, such as night sleep latency have not been commonly investigated to determine the relationship between whether related to the risk of hypertension. According to our knowledge, few studies have systematically investigated the relationship between night sleep latency and the risk of hypertension, as well as related sleep factors. This is the first study to systematically show the association between night sleep latency and the risk of hypertension, as well as hypertension-related sleep factors by gender in the Chinese population. Current results of our study showed that longer sleep latency was found in both men and women with hypertension and was independently positively associated with the risk of hypertension. These findings of our study were supported by several previous studies. A cross-sectional study based on the elderly population of Jino nationality suggested that sleep latency (OR = 2.98, 95% CI: 1.52–5.86) had a positive correlation with hypertension.^[39]^ A study with a 7-year follow-up reported that each 10-minute increase in sleep latency was associated with an 89% increase in hypertension risk (95% CI: 1.12–3.20).^[31]^ Another study also found that a longer sleep latency in women with hypertension.^[40]^

PSQI is a sleep assessment tool that investigates sleep factors to determine sleep patterns over the past 1 month.^[29]^ Compared with polysomnography, other sleep questionnaires, and clinical evaluations, the simultaneous and differential validity of the PSQI has been demonstrated.^[41]^ In general, a higher PSQI score indicates poor sleep quality. Until now, although several studies reported that PSQI is associated with hypertension,^[28,42,43]^ the relationship between global PSQI and hypertension remains controversial, which may be related to confounding factors such as age, gender, and region. Indeed, few reports showed the relationship between hypertension and PSQI components in the Chinese population. In the current study, we found a higher global PSQI score and its component score in hypertensive individuals. In addition, Spearman correlation analysis showed a positive correlation between night sleep latency and global PSQI scores in patients with hypertension. Further stratified analysis showed that longer night sleep latency had a higher global PSQI score, subjective sleep quality score, sleep latency score, daytime dysfunction score in men and women hypertension patients, but higher sleep duration score only in women hypertension patients. These results were similar to previous studies.^[23,44]^

In addition, our results also suggested that night sleep latency was positively correlated with sleep initiation time, was negatively correlated with sleep duration, sleep time on weekdays, and sleep time on weekends. Previous studies have demonstrated that sleep initiation time and sleep duration were associated with hypertension. A cohort study based on the Chinese population showed that longer night sleep duration and later sleep initiation time were related to the higher risk of hypertension, and night sleep duration and sleep initiation time may cumulatively increase the prevalence of hypertension in men.^[23]^ Indeed, according to previous reports, shorter night sleep duration appears to be more strongly associated with hypertension risk.^[45,46]^ Our results showed that night sleep duration was significantly shorter in hypertensive patients and night sleep latency was negatively correlated with sleep duration. Sleep time on weekdays and sleep time on weekends reflect people’s sleep conditions on weekdays and weekends respectively. In our study, we observed that men with hypertension had shorter night sleep time on both weekdays and weekends, which may be associated with curtailed sleep in favor of other leisure, social, or work-related activities. These results indicated that weekly sleep time shorten, possibly contributing to circadian misalignment, may be associated with hypertension risk.

In the present study, we adjusted for several confounding factors that might be relevant, including age, educational levels, BMI, smoking, drinking, sitting time on weekdays, sitting time on weekends, sleep time on weekdays, sleep time on weekends, night sleep duration, sleep initiation time, sleep latency, PSQI score, subjective sleep quality score, sleep latency score, sleep duration score, habitual sleep efficiency score, sleep disturbance score, use of sleep medication score, daytime dysfunction score. It is well known that age is an uncontrollable confounding factor, generally, the average sleep latency of adults increases with age, and older people are more likely to suffer from sleep.^[47]^ Several studies have reported that smoking can increase sleep latency in both men and women.^[48,49]^ Sahlin et al^[40]^ reported that women with alcohol dependency had longer sleep latency. In addition, other factors, including night-shift status, daytime sleepiness, sleep apnea or restless legs syndrome, and periodic movement disorders had been ruled out. Our results suggested that an independent positive correlation between night sleep latency and hypertension in both men and women after adjustment for confounding factors. Although current results cannot fully elucidate causation, our cross-sectional design suggested that the association between hypertension and longer night sleep latency was significant in both men and women, even after adjusting for all confounders. Unfortunately, we are unable to determine the mechanism of this relationship. However, it more and more appears that long latency to sleep and other sleep-related factors may contribute to the development and maintenance of hypertension through activation of sympathetic nervous system and hypothalamus–pituitary–adrenal axis, pro-inflammatory response, and endothelial dysfunction.^[15,50]^ Conversely, some scholars also proposed the hypothesis that hypertension, as a chronic stressor, may lead to the disruption of sleep homeostasis,^[51]^ such as the tension and anxiety caused by physical discomfort. Therefore, further investigation is needed to confirm the causality and potential mechanism of these findings.

## 5. Limitations

The current study had several limitations. First, it was a cross-sectional design based on a small sample, and the causality of night sleep latency with hypertension can’t be determined. Second, although we used self-reported sleep evaluations, the objective measurement of sleep quality is worth considering. Third, a 24-hour blood pressure monitoring blood pressure measurement seemed to be more accurate,^[52]^ which was not performed in our protocol. Fourth, participants’ psychological factors such as anxiety and depression status were not assessed, which may have confounded the current results. Fifth, the association between sleep latency and blood pressure was not assessed by age. In addition, some potential confounding factors, including family history, eating habits, daytime sleepiness, sleep apnea, restless legs syndrome, and periodic movement disorders were not be controlled. Lastly, we were unable to control for the confounding effects of light environment, location, and seasonal changes in blood pressure measurements because of the multicenter, long-period design of the study. Nevertheless, this study did provide a new perspective on the pathology of hypertension. This was one of the few studies to systematically investigate the relationship between night sleep latency and hypertension, as well as related sleep factors by gender in the Chinese population. It would be interesting to investigate the correlation between night sleep latency and blood pressure in future research.

## 6. Conclusions

In conclusion, the present findings show that hypertension is associated with long night sleep latency in both men and women. We report herein that long sleep latency may increase the risk of hypertension along with other sleep-related factors. More importantly, longitudinal studies with larger sample sizes are necessary to be conducted to further explore the causality and pathological mechanisms in the future.

## Acknowledgements

I would like to express my special thanks to my partners and our funding agency for the encouragement and support they gave me during this study.

## Author contributions

Huachen Jiao was the main coordinator of the project and was responsible for the study design. Xia Zhong and Fuyue Gou drafted the manuscript of the present paper. Dongsheng Zhao and Jing Teng involved in the supervising of data collection and stratification. Xia Zhong and Fuyue Gou contributed to data assembly and analysis. All authors contributed intellectually to this manuscript and have approved this final version.

**Conceptualization:** Xia Zhong, Fuyue Gou.

**Data curation:** Fuyue Gou, Dongsheng Zhao.

**Formal analysis:** Xia Zhong, Dongsheng Zhao.

**Funding acquisition:** Huachen Jiao.

**Investigation:** Fuyue Gou, Dongsheng Zhao.

**Methodology:** Xia Zhong, Jing Teng.

**Project administration:** Huachen Jiao.

**Resources:** Fuyue Gou, Huachen Jiao.

**Software:** Xia Zhong, Dongsheng Zhao.

**Supervision:** Jing Teng.

**Validation:** Jing Teng.

**Visualization:** Huachen Jiao.

**Writing – original draft:** Xia Zhong.

**Writing – review & editing:** Huachen Jiao.
